# Comparative metabolomics identifies enhanced ursane-type triterpenoids and antioxidant capacity in *Actinidia arguta* ‘Danyang’ kiwifruit

**DOI:** 10.3389/fpls.2026.1771986

**Published:** 2026-02-16

**Authors:** Mingzheng Duan, Jieyu Chang, Can Liang, Genxin Yang, Yuanqiao Li, Kaifeng Li, Xiande Duan, Lei Liu, Shunqiang Yang, Muhammad Junaid Rao

**Affiliations:** 1 Advanced Institute of Ecological Agriculture and Biodiversity on the Yunnan-Guizhou Plateau, Zhaotong University, Zhaotong, China; 2 State Key Laboratory for Development and Utilization of Forest Food Resources, Zhejiang A&F University, Hangzhou, China

**Keywords:** *Actinidia arguta*, antioxidant capacity, kiwifruit, metabolomics, terpenoid diversity

## Abstract

Chemical diversity is crucial for plant ecological adaptation and nutritional value. In kiwifruit, secondary metabolites such as terpenoids influence key traits such as flavor and nutraceutical properties, however, their diversity across different species and cultivars remains poorly characterized. This study employed UPLC-MS/MS-based metabolomics to profile terpenoids in five varieties representing both *Actinidia chinensis* (cultivars ‘Guichang’, GC; ‘SunGold’, SG; and a wild type, WL) and *Actinidia arguta* (cultivars ‘Maolvfeng’, LC; ‘Danyang’, DY). We identified 309 terpenoids, revealing profound diversity. Notably, the commercial *A. chinensis* ‘SunGold’ (SG) and *A. arguta* ‘Danyang’ (DY) exhibited the highest total terpenoid content, while SG uniquely possessed a soluble sugar content 2.4 to 6 times greater than other varieties. Both varieties also showed the strongest antioxidant capacity (455.20 and 438.91 µg TE/g FW, respectively), suggesting their superior nutraceutical potential. Multivariate analysis confirmed distinct terpenoid fingerprints, with DY enriched in ursane-type triterpenes e.g., pomolic acid, 1-oxo-siaresinolic acid, camellisin B, and siegesbeckic acid, while SG was uniquely abundant in seco-iridoids like geniposide (442-fold higher than WL). Within *A. chinensis*, the cultivated ‘Guichang’ (GC) showed a significant suppression of most triterpenoids compared to its wild relative (WL), demonstrating a possible breeding or selection effect. Our findings demonstrate that terpenoid profiles are likely influenced by genetic background, both at the species and cultivar level, providing a metabolic roadmap for the targeted breeding of kiwifruit with optimized health, nutraceutical, and sensory properties.

## 1 Introduction

Kiwifruit (genus *Actinidia*) has become a globally valued fruit, prized not only for its vibrant green flesh, tangy-sweet flavor, and distinctive texture but also for its significant nutritional and health-promoting properties (Rakha et al., 2025). The commercial success of cultivars like ‘SunGold’ (from *Actinidia chinensis*) has stimulated the cultivation and study of a wide array of varieties, each with unique genetic backgrounds and geographical origins (Ferguson et al., 2023). This diversity is not only morphological (such as variations in size, shape, and skin color) but is intensely reflected in their biochemical composition, making it a key target for modern breeding programs. Beyond primary metabolites like sugars and vitamins, kiwifruits are rich in secondary metabolites. These include polyphenols, flavonoids, anthocyanin, proanthocyanidins, and terpenoids, which are crucial determinants of their sensory quality, antioxidant capacity, and potential bioactivity (Yu et al., 2020; Jia et al., 2022). Therefore, understanding metabolic diversity across kiwifruit genotypes, including distinct species such as *A. chinensis* and *A. arguta*, is fundamental for genetic improvement and the selection of superior cultivars with enhanced nutritive value and potential for functional food development (Nazir et al., 2024).

Secondary metabolites are phytochemicals that play vital roles in plant defense, communication, and adaptation (Rao and Zheng, 2025; Rao et al., 2024). In the context of human consumption, these compounds are increasingly recognized for their contributions to health beyond basic nutrition (Rao et al., 2025). In kiwifruit, current research has largely focused on quantifying well-known attributes such as vitamin C content, antioxidant activity, and the composition of primary sugars and dietary fiber (Zhang et al., 2020). More recently, studies have begun to profile specific secondary metabolite classes, particularly phenolic compounds, linking them to the fruit’s antioxidant and anti-inflammatory properties (Li et al., 2024; Moysidou et al., 2024, 2025). However, a significant research gap remains: the comprehensive characterization of other major phytochemical groups, especially terpenoids, across diverse kiwifruit varieties and species is notably lacking (Li et al., 2024). Furthermore, the intricate relationship between genetic background, geographical origin, and the biosynthesis of these specialized metabolites is poorly understood. This knowledge gap limits the efficiency of breeding strategies aimed at enhancing these valuable traits.

Terpenoids, also known as isoprenoids, constitute one of the largest and most structurally diverse families of natural products. They are classified based on the number of isoprene (C5) units they contain, encompassing monoterpenes (C10), sesquiterpenes (C15), diterpenes (C20), triterpenes (C30), and tetraterpenes (C40, such as carotenoids) (Tholl, 2015; Lu et al., 2016; Holopainen et al., 2025). In plants, terpenoids serve essential ecological functions. Volatile mono- and sesquiterpenes attract pollinators, while non-volatile tri- and diterpenes often provide defense against herbivores and pathogens (Holopainen et al., 2025). For humans, their importance is immense. Monoterpenes and sesquiterpenes are the primary constituents of essential oils, dictating the aroma profiles of fruits like citrus, mango, and grapes, which directly influence consumer preference (Abbas et al., 2023). Triterpenoids, frequently found in their glycosylated forms as saponins, are investigated for a wide range of bioactivities, including anti-inflammatory, antimicrobial, antiviral, and anticancer properties (El-Dawy et al., 2022; Liu et al., 2022; Malik and Mandal, 2024). Despite their known significance for flavor and health, the terpenoid diversity of kiwifruit remains poorly characterized. Systematic profiling studies across different varieties and species are notably limited, thereby overlooking a vast reservoir of compounds relevant for cultivar development.

This identified research gap underscores the need for a detailed, comparative investigation of the kiwifruit terpenoid metabolome. While recent studies have made significant progress such as identifying specific terpene synthases (TPS) like AcTPS1b responsible for key flavor compounds such as 1,8-cineole and characterizing the small but functionally redundant TPS family in kiwifruit genotypes (Zeng et al., 2020; Wang et al., 2023). Building on this foundation, critical questions remain unanswered: How does the terpenoid profile vary among commercially important and wild kiwifruit varieties from different genetic backgrounds? To what extent does genetic background influence the accumulation of specific terpenoid classes? What is the correlation between terpenoid composition and established functional properties like antioxidant capacity? Addressing these questions is crucial for informing precision breeding for enhanced kiwifruit quality.

Therefore, this study employs a widely targeted UPLC-MS/MS metabolomics approach to comprehensively profile terpenoid diversity in five distinct kiwifruits, representing both *Actinidia chinensis* and *Actinidia arguta*, including commercial cultivars and a wild type. By integrating this metabolic data with analyses of sugar content and antioxidant activity, we aim to decode the varietal-specific terpenoid fingerprints, identify key differing metabolites, and elucidate the relationship between terpenoid abundance and functional food properties. This research will provide a valuable scientific foundation and identify metabolic markers to support the genetic improvement of kiwifruit and the development of high-value cultivars with optimized flavor and health-promoting attributes through targeted breeding strategies.

## 2 Materials and methods

### 2.1 Fresh sample preparation of kiwifruit

Fresh kiwifruit samples from five varieties were selected: GC (*Actinidia chinensis* ‘Guichang’, Zhaotong), WL (*Actinidia chinensis*, Wild Kiwifruit, Zhaotong), LC (*Actinidia arguta* ‘Maolvfeng’, Dandong), DY (*Actinidia arguta*, Danyang Flat Kiwifruit, Dandong), and SG (*Actinidia chinensis* ‘SunGold’, New Zealand). Fruits from all varieties were harvested at a commercially ripe stage. For this comparative study, all post-harvest handling and sampling were performed under identical conditions. The fruits were gently washed with clean water to remove surface soil and impurities, then air-dried on sterile gauze or absorbent paper.

All grinding tools, including mortar and pestle, were wrapped in kraft paper and sterilized using an autoclave at 121°C for 20–30 minutes. Using a sterile scalpel, the skin of each fruit was carefully removed, and only the flesh was used for subsequent analysis. For each variety, flesh from multiple fruits was combined and ground into a homogeneous mixture to create one biological replicate. Three independent biological replicates were prepared per variety. A portion of the ground pulp was tightly wrapped in aluminum foil to form spherical samples, which were then placed into pre-cooled centrifuge tubes and appropriately labeled.

The samples were rapidly frozen by immersing the tubes in liquid nitrogen for 2–5 minutes to ensure complete solidification. Finally, all samples were stored together in a –80°C ultra-low temperature freezer for further metabolic analysis.

### 2.2 Determination of reducing sugar content

The reducing sugar content in kiwifruit samples was quantified using the 3,5-dinitrosalicylic acid (DNS) method (Lam et al., 2021). Approximately 0.1 g of fruit tissue from each variety was homogenized to 0.8 mL of 80% ethanol using an ice bath, and the mixture was transferred to a centrifuge tube. The final volume was adjusted to 1.5 mL with 80% ethanol.

The tubes were sealed and incubated in a water bath at 50°C for 20 minutes, mixing every 2 minutes. After cooling, the volume was readjusted if necessary, and the extracts were centrifuged at 12,000 rpm for 10 minutes. The supernatant was collected for analysis. A 100 µL aliquot of the supernatant was mixed with 100 µL of DNS reagent in a microtube, heated at 95°C for 10 minutes, and then cooled rapidly. After adding 1 mL of distilled water, 200 µL of the mixture was transferred to a 96-well plate, and the absorbance was measured at 500 nm using a microplate reader. A standard curve was generated using glucose standards, and the reducing sugar content was calculated according to the formula:


Reducing sugar (mg/g)=1.496×((ΔA+0.0324))/W×D

where ΔA is the corrected absorbance, *W* is the sample weight (g), and *D* is the dilution factor.

### 2.3 Determination of soluble sugar content

The soluble sugar content was determined using the anthrone-sulfuric acid method (Liu et al., 2025). Briefly, approximately 0.1 g of fruit tissue from each variety was homogenized in 80% ethanol, with the final extract volume adjusted to 1.5 mL. The extracts were incubated at 50°C for 20 minutes, centrifuged, and the supernatant was collected. For the assay, 25 µL of the supernatant was mixed with 75 µL of distilled water and 250 µL of anthrone reagent. Then, 250 µL of concentrated sulfuric acid was added slowly to the mixture. The tubes were heated at 95–100°C for 10 minutes, cooled, and the absorbance was measured at 620 nm using a microplate reader. A standard curve was generated using glucose, and the soluble sugar content was calculated according to the following formula:


Soluble sugar (mg/g)=0.718×((ΔA+0.0103))/W×D

where ΔA is the corrected absorbance, *W* is the sample fresh weight (g), and *D* is the dilution factor.

### 2.4 Determination of DPPH radical scavenging activity

The DPPH (1,1-diphenyl-2-picrylhydrazyl) radical scavenging activity was measured to evaluate the antioxidant capacity of the kiwifruit samples (Wojtunik et al., 2014). Approximately 0.1 g of kiwifruit sample was ground, passed through a 40-mesh sieve, and extracted with 1 mL of 80% methanol using ultrasonic assistance at 60°C for 30 minutes. After centrifugation at 12,000 rpm for 10 minutes, the supernatant was collected. For the assay, 150 µL of the extract was mixed with 150 µL of DPPH working solution. The mixture was incubated in the dark at 25°C for 30 minutes, centrifuged, and the absorbance of the supernatant was measured at 517 nm. A standard curve was prepared using Trolox, and the radical scavenging activity was calculated as follows:


DPPH scavenging capacity (μg Trolox/g FW)=0.351×((Scavenging % −0.7084))/W×D

where *W* is the sample fresh weight (g), and *D* is the dilution factor.

### 2.5 Profiling of terpenoids in kiwifruits

A widely targeted metabolomic approach was employed to profile chemical diversity of terpenoids across the five kiwifruit varieties using an Ultra Performance Liquid Chromatography-Tandem Mass Spectrometry (UPLC-MS/MS) platform (Wuhan Metware Biotechnology Co., Ltd.). Three independent biological replicates per variety were analyzed. The analysis was performed on a system consisting of an ExionLC™ AD UPLC (SCIEX) for chromatographic separation coupled to a triple quadrupole-linear ion trap (QTRAP^®^ 6500+) mass spectrometer (SCIEX) for detection. Fresh fruit samples were freeze-dried, ground to a powder, and 30 mg of the powder was extracted with 1500 μL of a pre-cooled 70% methanol solution containing internal standards (CAS:14091-11-3; 2-chlorophenylalanine, 1PPM mg/L) for quality control and relative quantification. The extracts were vortexed, centrifuged, and the supernatant was filtered through a 0.22 μm membrane for analysis. A pooled quality control (QC) sample, prepared by combining equal aliquots from all individual extracts, was injected at regular intervals, to monitor instrument stability and correct for signal drift. Chromatographic separation was performed on an Agilent SB-C18 column (2.1 mm × 100 mm, 1.8 μm) using a gradient of water (A) and acetonitrile (B), both containing 0.1% formic acid, at a flow rate of 0.35 mL/min and a column temperature of 40°C.

Mass spectrometric detection was carried out on a triple quadrupole (QQQ) instrument equipped with an electrospray ionization (ESI) source operating in both positive and negative modes. Data acquisition was performed in Multiple Reaction Monitoring (MRM) mode, on a triple quadrupole mass spectrometer, with method parameters established from the Metware Database (MWDB). Data were acquired in both positive and negative electrospray ionization modes (ESI+/-) with ion spray voltages of 5500 V and -4500 V, respectively, a source temperature of 500°C, and gas parameters set at 50 psi (GSI), 60 psi (GSII), and 25 psi (curtain gas). For each metabolite, unique MRM transitions were established by optimizing the declustering potential and collision energy to select a specific precursor ion in the first quadrupole (Q1) and its most abundant and characteristic product ion in the third quadrupole (Q3), with a medium setting for collision-induced dissociation nitrogen gas. A scheduled MRM algorithm was employed, monitoring specific metabolite transitions only during their predefined retention windows to ensure sufficient data point acquisition across chromatographic peaks for precise relative quantification based on integrated peak areas, normalized to the internal standards and sample weight (Chen et al., 2013; Rao et al., 2022). The molecular formula, precursor (Q1) and product (Q3) ions for MRM transitions, molecular characteristics, ionization mode, confidence level of identification, and CAS registry number of all detected terpenoids are represented (**Supplementary Table S1**). Metabolite identification and qualification data were achieved by matching the acquired MS/MS spectra and retention times against the proprietary Metware Database (MWDB).

### 2.6 Statistical and bioinformatic analysis

Multivariate statistical analyses were conducted to explore the metabolic variation among the five kiwifruit varieties. The relative quantification data from the metabolomic profiling were initially standardized using unit variance scaling. Principal component analysis (PCA) was performed to visualize the clustering of the kiwifruit samples. Variable Importance in Projection (VIP) scores from the OPLS-DA model were calculated, and metabolites with a VIP score > 1.0 were considered significant contributors to group discrimination (Thévenot et al., 2015).

Differential metabolites between comparison groups were identified by applying a dual-threshold criterion: a fold change of |log2FC| ≥ 1.0 and a statistically significant p-value (< 0.05) from a student’s t-test, adjusted for multiple comparisons using the false discovery rate (FDR) (Colquhoun, 2014). Hierarchical cluster analysis (HCA) was performed on the normalized data to visualize the accumulation patterns of metabolites across all kiwifruit samples. All statistical analyses and visualizations, including PCA and HCA, were performed using R software (version 4.1.2) (Chanana et al., 2020). For the analysis of biochemical traits (e.g., antioxidant capacity, sugar content), Fisher’s Least Significant Difference (LSD) test was conducted using Statistix 8.1, with significance defined at p< 0.05. EVenn web-based online (https://www.bic.ac.cn/EVenn/#/) free platform was employed to perform Venn analysis (Yang et al., 2024).

## 3 Results

### 3.1 Comparative terpenoid profiling reveals significant varietal differences in kiwifruit

Liquid chromatography tandem mass spectrometry (LC-MS/MS) analysis revealed a diversity of terpenoids across the five kiwifruit varieties studied. In total, 309 distinct terpenoid compounds were identified and divided into major terpenoid classes such as 82 triterpenes, 79 sesquiterpenoids, 75 monoterpenoids, 57 diterpenoids, 10 terpene, and 6 triterpene saponins, underscoring the complex phytochemical diversity of kiwifruit (**Supplementary Table S1**).

**Figure 1:**
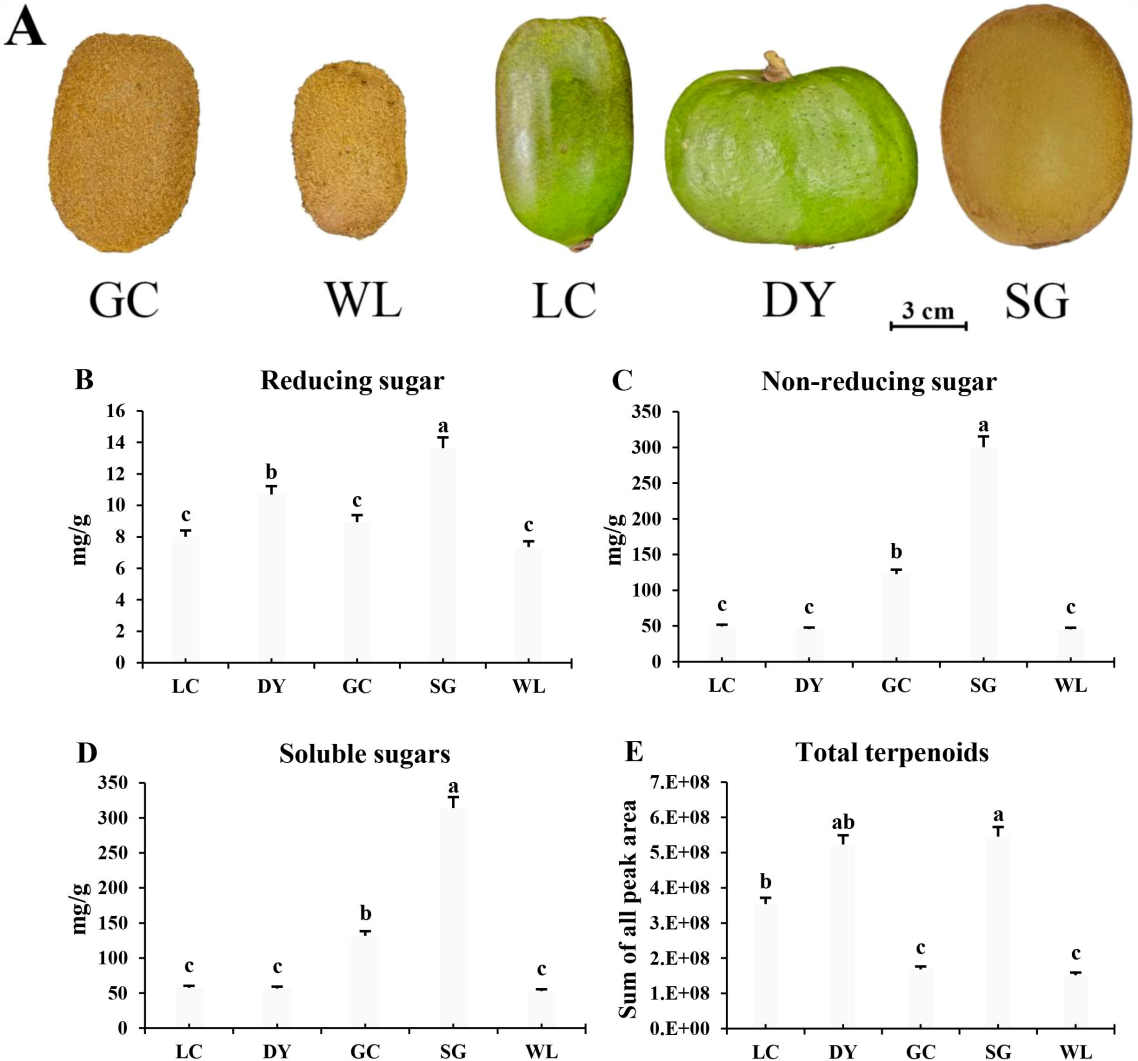
Morphological traits, sugar content, and total terpenoid abundance across five kiwifruit varieties. **(A)** Representative fruit morphology of the five varieties. **(B)** Reducing sugar content. **(C)** Non-reducing sugar content. **(D)** Total soluble sugar content. **(E)** Total terpenoid content, based on the cumulative peak area from LC-MS/MS. Data in **(B-E)** are presented as mean ± standard error (SE) n=3. Lowercase letters (a, b, c) indicate significant differences by Fisher’s LSD test (P< 0.05). LC (*Actinidia arguta* ‘Maolvfeng’, Dandong), DY (*Actinidia arguta*, Danyang flat kiwifruit, Dandong), GC (*Actinidia chinensis* ‘Guichang’, Zhaotong), SG (*Actinidia chinensis* ‘SunGold’, New Zealand), and WL (*Actinidia chinensis*, Wild type, Zhaotong).

The visual analysis of the five kiwifruit varieties reveals significant morphological diversity in terms of fruit outer skin color, size, and shape (**Figure 1A**). The GC and WL fruits are oval in shape. The LC and DY are intense green outer skin, however, the LC fruit is oval while the DY fruit is flat. The SG is brown in color. The soluble sugar content results revealed that the SG fruits exhibited higher reducing sugar (13.64 mg/g), non-reducing sugar (300.4 mg/g), and total soluble sugar content (314.04 mg/g), which was approximately 2.4 to 6 times greater than the other varieties (**Figures 1B–D**). The GC variety showed the second-highest level (131.72 mg/g), while the remaining three varieties (LC, DY, and WL) showed considerably lower and relatively similar total sugar contents, ranging from 52.76 to 57.47 mg/g. Similar pattern was observed in non-reducing sugars, which constituted the dominant fraction of soluble sugars in all varieties, with SG and GC again displaying the highest concentrations. Reducing sugar content was less variable but was also highest in the SG variety. This stark contrast highlights the likely impact of genetic background on sugar accumulation, with the commercial SG variety indicating a superior sweetness potential.

The quantitative analysis, based on the sum of all chromatographic peak areas, revealed significant varietal differences in the total terpenoid content (**Figure 1E**). The SG variety from New Zealand and the DY flat kiwifruit exhibited the highest overall terpenoid abundance (**Figure 1E**). In contrast, the WL and the GC variety, both from Zhaotong, displayed substantially lower total terpenoid contents (**Figure 1E**). These findings suggest that genetic background (variety) and geographical origin along with possible environmental and agronomic factors are key factors influencing the terpenoid biosynthetic pathway in kiwifruit.

### 3.2 Principal component analysis of terpenoid among kiwifruit varieties

**Figure 2:**
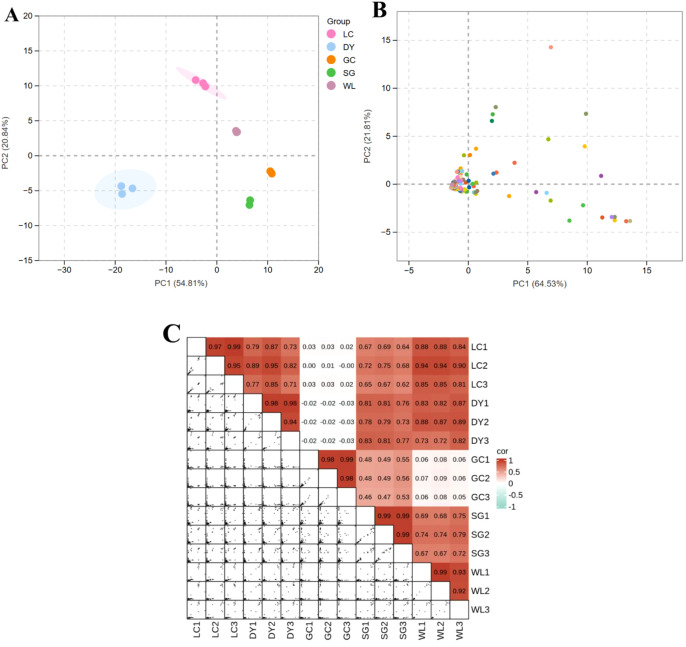
Principal Component Analysis (PCA) and correlation analysis of Kiwifruit terpenoid profiles. **(A)** PCA score plot of samples (variety-wise). Each point represents one biological replicate. **(B)** Compound-wise PCA loading plot of terpenoid profiles. Each colored dot represents a unique terpenoid compound, assigned a code (TR1 to TR199). The color coding and corresponding full chemical names for these compounds are provided in **Supplementary Figure S1** and **Supplementary Table S2**, respectively. **(C)** Pearson correlation matrix of terpenoid profiles across kiwifruit varieties. Positive correlation (red) and low or negative correlation (white or green). LC (*Actinidia arguta* ‘Maolvfeng’, Dandong), DY (*Actinidia arguta*, Danyang flat kiwifruit, Dandong), GC (*Actinidia chinensis* ‘Guichang’, Zhaotong), SG (*Actinidia chinensis* ‘SunGold’, New Zealand), and WL (*Actinidia chinensis*, Wild type, Zhaotong).

Principal Component Analysis (PCA) of the terpenoid profiles revealed clear separations among the five kiwifruit varieties, indicating that their overall terpenoid compositions are distinct and characteristic (**Figure 2A**). The first two principal components (PC1 and PC2) explained a cumulative 75.65% of the total variance (54.81% and 20.84%, respectively). The clear separation indicates that each variety possesses a unique and characteristic terpenoid composition. Compound-wise PCA showed significant variation among terpenoids, the PC1 accounted for 64.53% while the PC2 accounted for 21.81% of variation (**Figure 2B**). Most of the terpenoids were clustered near the intersection point of x-axis and y-axis, while a few numbers of compounds scattered across the PCA-plot, showing dissimilar characteristics among terpenoids (**Figure 2B**). This structured grouping demonstrates that the terpenoid metabolome is a strong chemotaxonomic marker capable of distinguishing kiwifruit genotypes. Correlation analysis showed a strong positive correlation in terpenoid composition among biological replicates of the same variety (**Figure 2C**). Most intra-variety correlation coefficients exceeded 0.90, indicating high reproducibility and a homogeneous terpenoid profile for each genotype. In contrast, inter-variety comparisons generally showed low or negative correlations, indicating that each kiwifruit variety possesses a distinct terpenoid fingerprint.

### 3.3 Varietal differences in terpenoid profiles revealed by hierarchical clustering analysis

**Figure 3:**
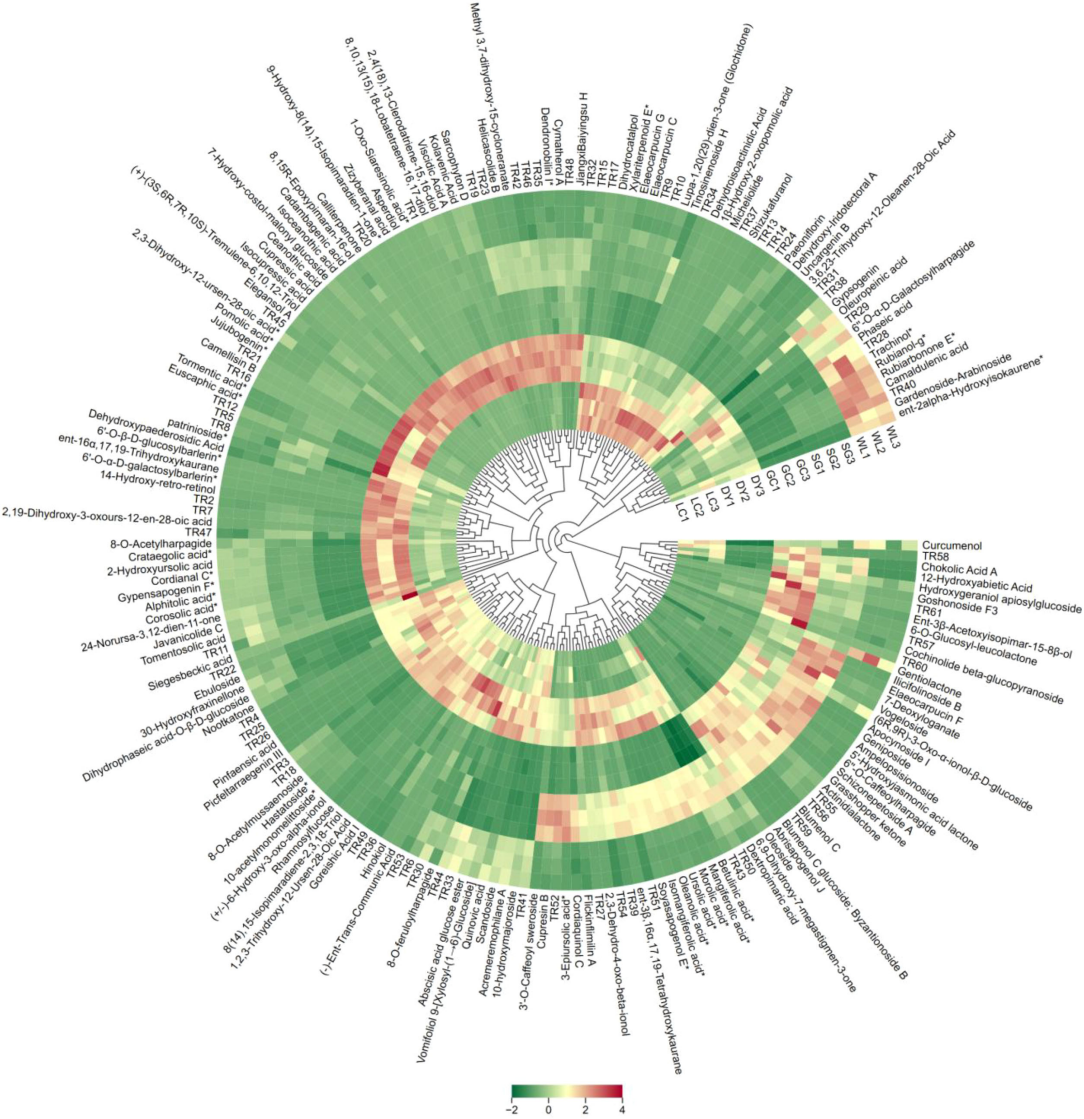
Hierarchical Clustering Analysis (HCA) of significantly altered terpenoids (row Z-score normalized) across five kiwifruit varieties. Each row represents terpenoid compound, and each column represents a kiwifruit variety. The red denotes higher abundance, green denotes lower abundance, and yellow represents average levels. The abbreviated terpenoids (TR1-TR61) full names are signified in **Supplementary Table S2**. LC (*Actinidia arguta* ‘Maolvfeng’, Dandong), DY (*Actinidia arguta*, Danyang flat kiwifruit, Dandong), GC (*Actinidia chinensis* ‘Guichang’, Zhaotong), SG (*Actinidia chinensis* ‘SunGold’, New Zealand), and WL (*Actinidia chinensis*, Wild type, Zhaotong).

Hierarchical Clustering Analysis (HCA) was performed on the 198 terpenoids that were identified as significantly altered (VIP > 1.1). The heatmap (**Figure 3**) revealed clear varietal-specific differences in terpenoid accumulation. The HCA showed that DY variety was high in abundance of ursane-type triterpenes, such as 2,3-Dihydroxy-12-ursen-28-oic acid, pomolic acid, and jujubogenin present than in other varieties (**Figure 3**). The WL and LC varieties formed a cluster largely due to their shared, high levels of oleanane-type triterpenes, such as alphitolic acid, gypensapogenin F, and crataegolic acid, which were consistently elevated in WL and LC compared to GC and SG. In contrast, the SG variety was separated by a unique combination of moderate to high levels of specific triterpenoids like ursolic acid, oleanolic acid, and asiatic acid. The cultivated GC consistently shows low concentrations of triterpenoids that were elevated in the other varieties. This general suppression of triterpenoid biosynthesis was a key factor in its clear separation from its wild (WL) and the other varieties. This HCA analysis determines that the terpenoid landscape is highly dependent on the kiwifruit genotype and origin.

### 3.4 Pairwise comparison of terpenoid profiles across different kiwifruits

**Figure 4:**
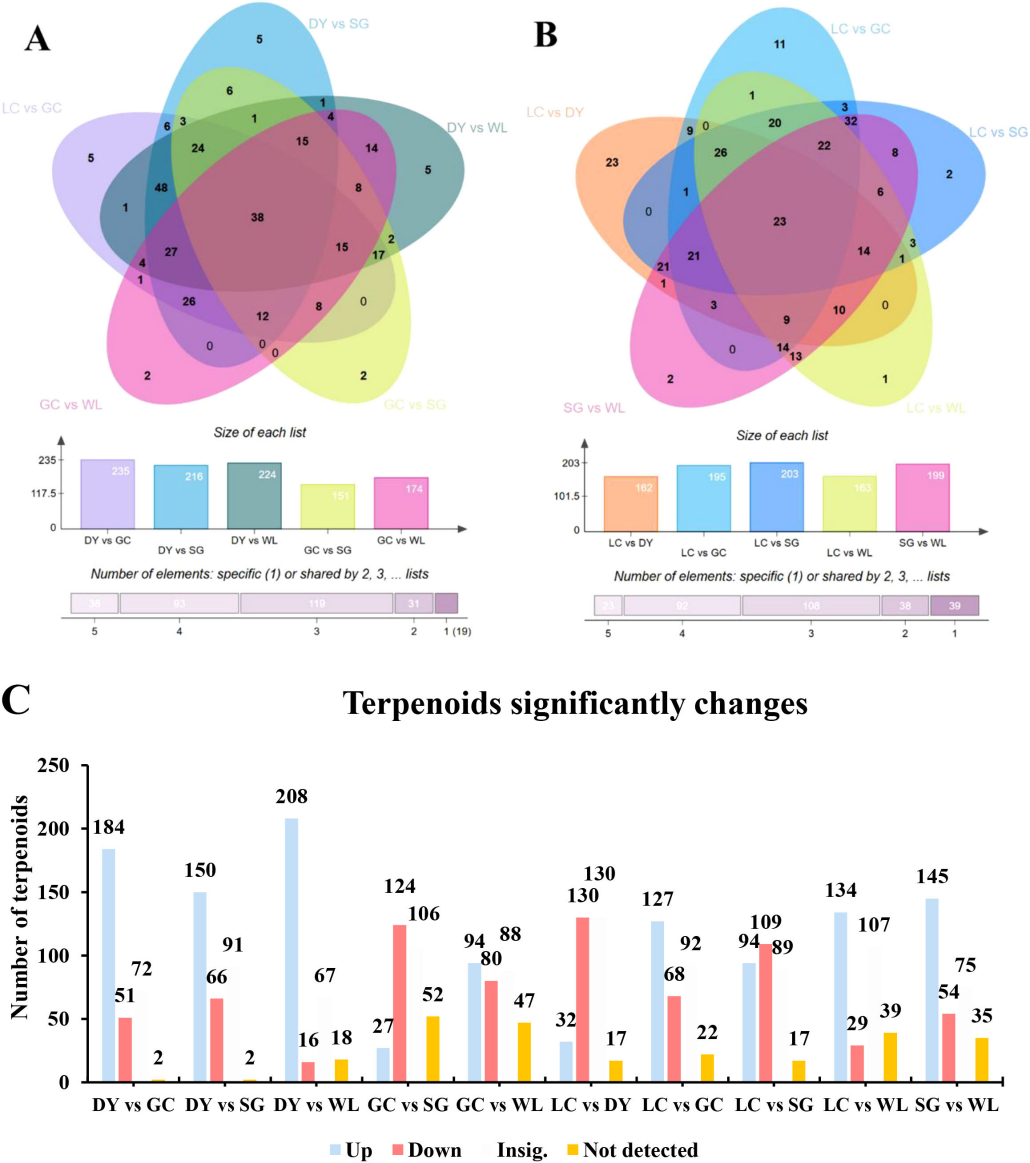
Comparative analysis of terpenoids across different comparison groups. **(A, B)** Venn diagram illustrates the number of unique and shared compounds among different comparison groups of five kiwifruit varieties (DY, SG, LC, GC, WL). **(C)** Pairwise comparison of significantly altered terpenoids. The significantly upregulated (Up), downregulated (Down), or not significantly changed (Insig.) for each varietal pair, based on a threshold of VIP > 1.0 and FC > 1.0 (**Supplementary Table S3**). LC (*Actinidia arguta* ‘Maolvfeng’, Dandong), DY (*Actinidia arguta*, Danyang flat kiwifruit, Dandong), GC (*Actinidia chinensis* ‘Guichang’, Zhaotong), SG (*Actinidia chinensis* ‘SunGold’, New Zealand), and WL (*Actinidia chinensis*, Wild type, Zhaotong).

The Venn diagram analysis revealed distinct profiles of terpenoids among different comparison groups of five kiwifruit varieties (DY, SG, LC, GC, WL) (**Figures 4A, B**). A total of 38 compounds were commonly present in all five comparison groups, the DY vs GC showed highest 235 number of identified compounds while the GC vs SG showed lowest 151 number of terpenoids (**Figure 4A**). Similarly, in second Venn diagram, a total of 23 compounds were commonly present in all five comparison groups, the LC vs SG showed highest 203 number of identified compounds followed by SG vs WL 199 while the LC vs DY showed lowest 162 number of terpenoids (**Figure 4B**). The comparison group LC vs DY revealed maximum number of 23 unique compounds followed by LC vs GC 11 (**Figure 4B**). This indicates that a set of compounds is common among different comparison groups, while each group possesses a unique terpenoid fingerprint, suggesting significant terpenoids diversity among kiwifruit varieties.

Pairwise comparison of terpenoid profiles were performed to evaluate the significantly altered (Variable Importance in Projection, VIP > 1.0 and fold-change, FC > 1) terpenoids among varieties (**Figure 4C**). The comparison between DY and WL showed the most pronounced variation, with 208 terpenoids significantly upregulated in DY and only 16 upregulated in WL. Similarly, comparisons involving the high-terpenoid varieties (DY and SG) against the low-terpenoid ones (GC and WL) consistently showed high number of upregulated metabolites. In contrast, the comparison between the two low-terpenoid varieties, GC and WL, showed a more balanced alteration (94 up in GC, 80 up in WL). Notably, the comparison between GC and SG revealed that 124 terpenoids were significantly higher in SG, while only 27 were higher in GC, highlighting the distinct phytochemical signature of the commercial ‘SunGold’ variety (**Figure 4C**). These results confirm that beyond the total content, the specific composition and abundance of individual terpenoids are highly variety specific.

### 3.5 Percentage abundance of major terpenoids among kiwifruit varieties

**Figure 5:**
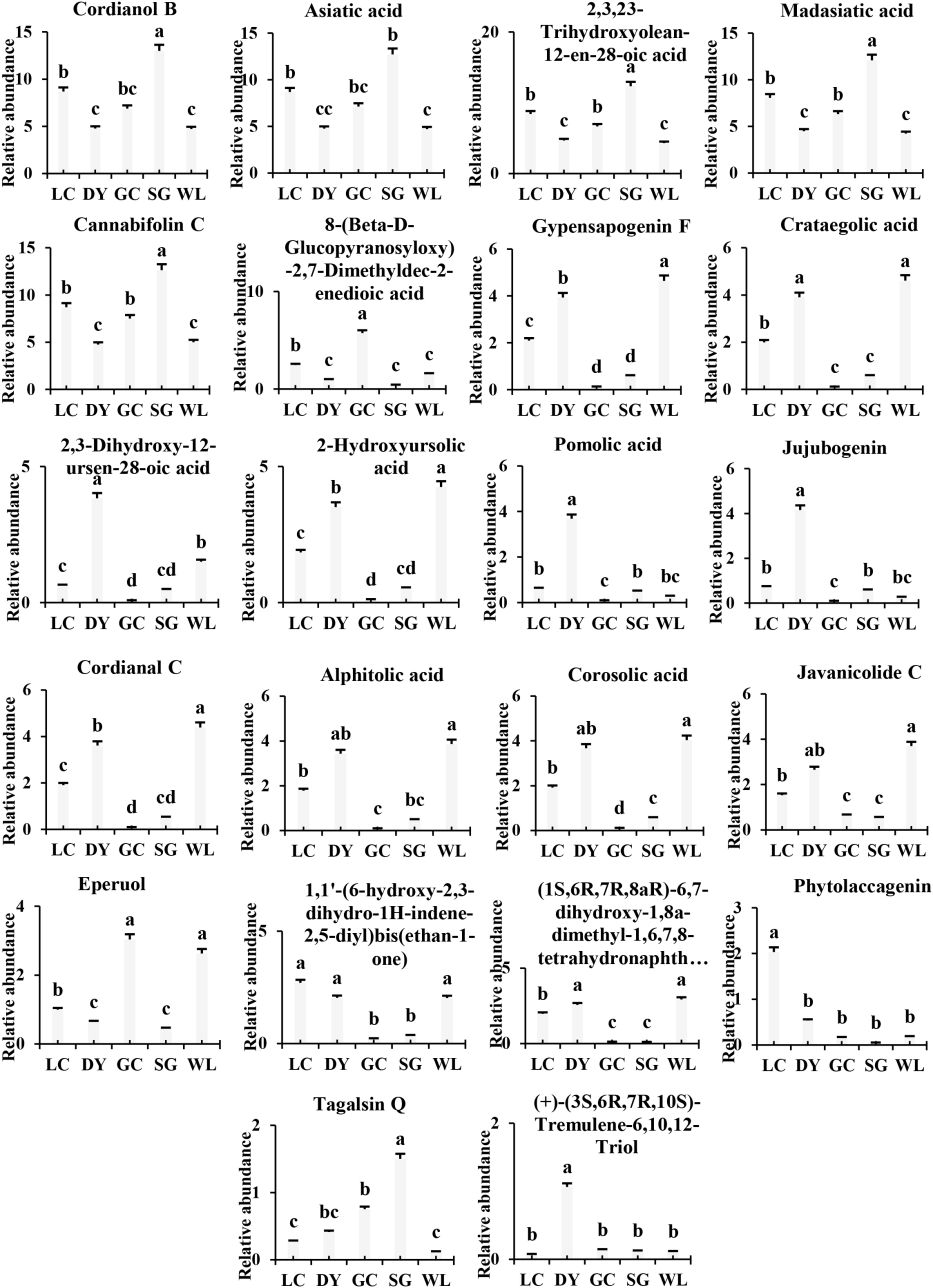
Percentage abundance of major terpenoid compounds in five kiwifruit varieties. Data are presented as mean values of biological replicates (n=3, bars represent ± SE) for each variety (**Supplementary Table S4**). Lowercase letters (a, b, c) indicate significant differences by Fisher’s LSD test (P< 0.05). LC (*Actinidia arguta* ‘Maolvfeng’, Dandong), DY (*Actinidia arguta*, Danyang flat kiwifruit, Dandong), GC (*Actinidia chinensis* ‘Guichang’, Zhaotong), SG (*Actinidia chinensis* ‘SunGold’, New Zealand), and WL (*Actinidia chinensis*, Wild type, Zhaotong).

The percentage abundance of 22 major terpenoids showed differences among kiwifruit varieties (**Figure 5**; **Supplementary Table S4**). The SG variety showed higher percentage abundance of specific triterpenic acids, such as cannabifolin C, asiatic acid, and madasiatic acid, which were about 1.5 to 2.7 times higher than other varieties. This suggests a distinct biosynthetic pathway favoring these compounds in the SG genotype. In contrast, the LC and DY varieties are rich in pentacyclic triterpenes such as gypensapogenin F, 2-hydroxyursolic acid, and alphitolic acid, though DY exhibited higher concentrations across these compounds. Notably, pomolic acid and its derivative, 2,3-dihydroxy-12-ursen-28-oic acid, were exceptionally abundant in the DY variety, serving as a key chemical signature (**Figure 5**).

The WL displayed diverse terpenoid profile, with high levels of alphitolic acid, gypensapogenin F, and javanicolide C, indicating a complex and potent terpenoid backbone. Its cultivated counterpart from the same region, GC, was starkly different, showing a general suppression of most triterpenic acids but a unique, high accumulation of 8-(Beta-D-Glucopyranosyloxy)-2,7-Dimethyldec-2-enedioic acid, a glycosylated terpenoid derivative. This obvious difference among the wild and cultivated types from the same geographical origin highlights the significant impact of possible breeding and selection effect on secondary metabolite production. Overall, the results showed that each kiwifruit variety possesses a unique terpenoid “fingerprint,” with implications for their sensory properties, nutritional value, and potential bioactivity.

### 3.6 Pairwise comparative analysis identifies varietal terpenoid markers

**Figure 6:**
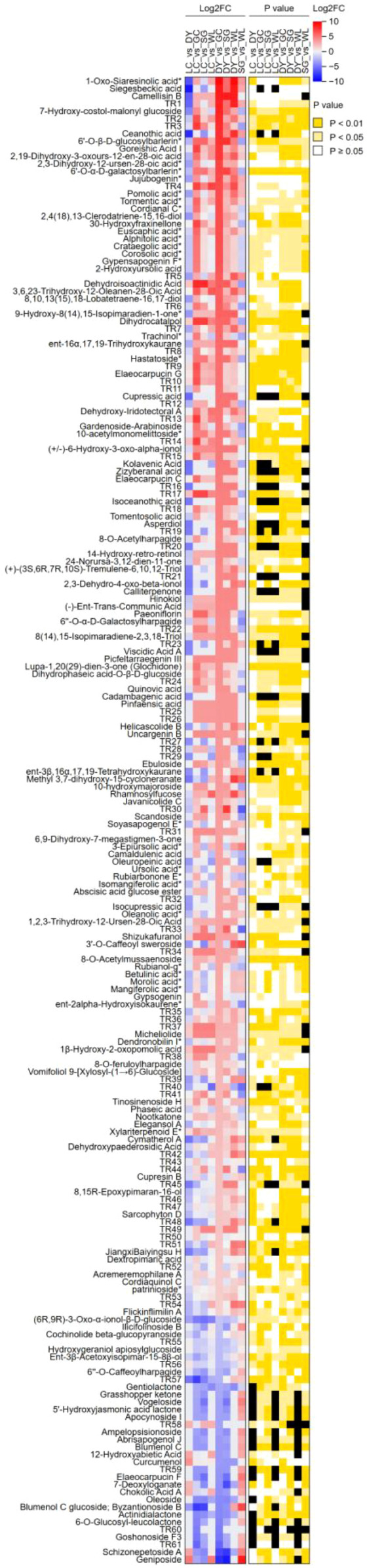
Heatmap of terpenoid compounds across five kiwifruit varieties. The heatmap visualizes the relative abundance (Log_2_Fold Change, left panel) and statistical significance (p-value, right panel) of terpenoids across eight pairwise comparisons between the varieties: LC (*Actinidia arguta* ‘Maolvfeng’, Dandong), DY (*Actinidia arguta*, Danyang flat kiwifruit, Dandong), GC (*Actinidia chinensis* ‘Guichang’, Zhaotong), SG (*Actinidia chinensis* ‘SunGold’, New Zealand), and WL (*Actinidia chinensis*, Wild type, Zhaotong). The color scale for Log_2_FC ranges from blue (downregulated) through white (no change) to red (upregulated). Log_2_FC and p-value of each compound are represented (**Supplementary Table S3**). The p-value intensity increases with color dark yellowness.

The quantitative analysis of terpenoid compounds revealed significant variations in their abundance across the sample groups. Several terpenoids demonstrated significant alteration, as indicated by their log2 Fold Change (log_2_FC) values, which were supported by statistically significant p-values (**Figure 6**). The varieties SG and DY showed significantly upregulation (Log_2_FC > 8) of most of the terpenoids compounds as compared to other three varieties. These results highlight that the terpenoid profile was distinctly modulated, with certain compounds being significantly upregulated or downregulated in specific comparisons.

**Figure 7:**
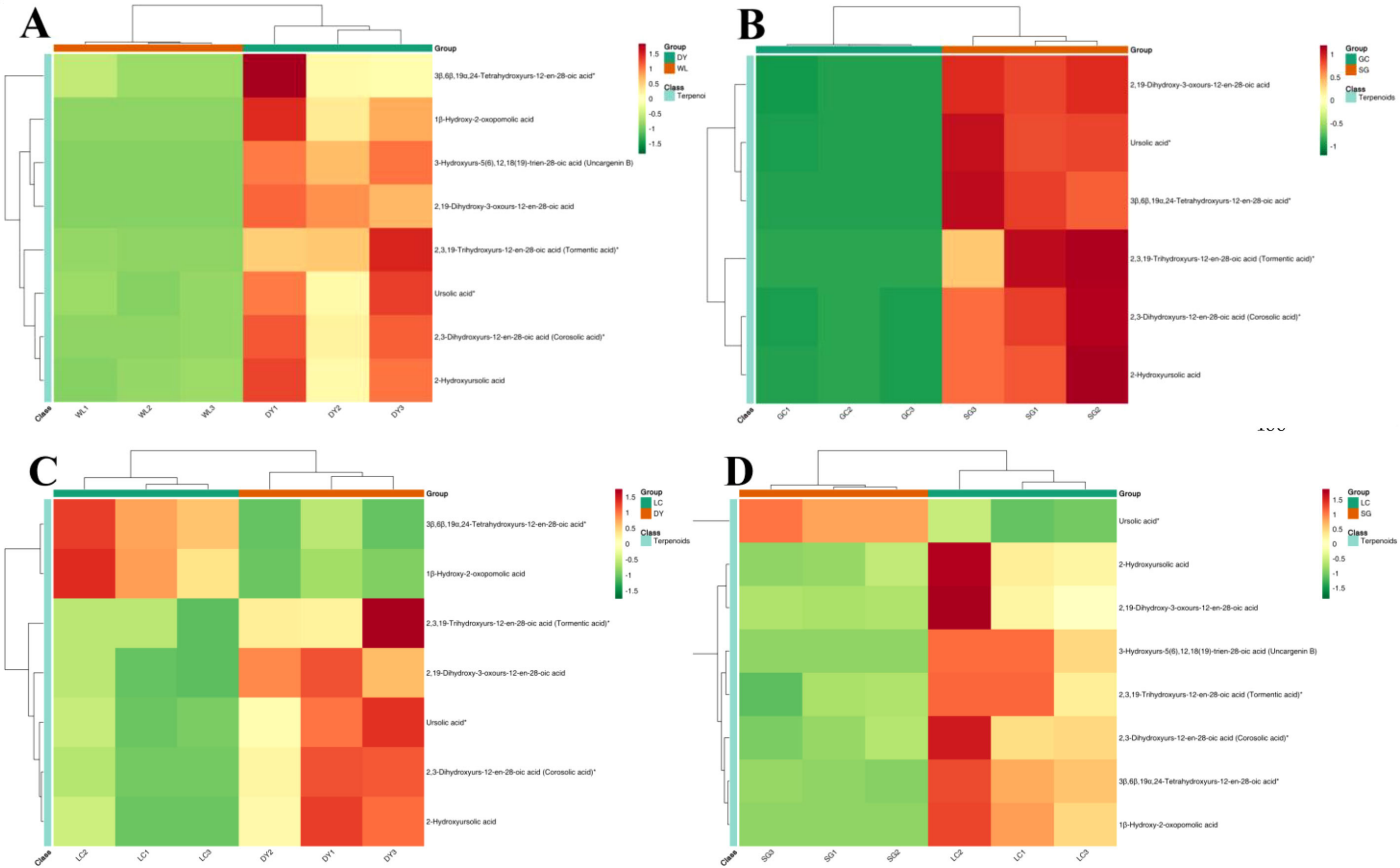
Differential abundance heatmaps of ursane-type triterpenoid acids in key kiwifruit variety comparisons. Heatmaps display the relative abundance (Z-score normalized) of selected ursane-type triterpenes that were most significantly altered in pairwise comparisons. Red indicates higher abundance, blue indicates lower abundance, and white represents average levels. **(A)** DY (Danyang, *A*. *arguta*) versus WL (Wild, *A*. *chinensis*). **(B)** GC (Guichang, *A*. *chinensis*) vs SG (‘SunGold’, *A*. *chinensis*). **(C)** LC (Maolvfeng, *A*. *arguta*) vs DY (Danyang, *A*. *arguta*). **(D)** LC (Maolvfeng, *A*. *arguta*) vs SG (‘SunGold’, *A*. *chinensis*). These visualizations underscore the distinct terpenoid fingerprints shaped by genotype and cultivation status.

To further visualize the specific terpenoids (ursane-type triterpenes) that were most significantly altered in key varietal comparisons, we performed differential abundance heatmaps (**Figure 7**). In the comparison between the high-terpenoid DY and the wild type (WL), nearly all analyzed ursane-type triterpenes were obviously abundant in DY, with compounds like 3β,6β,19α,24-tetrahydroxyurs-12-en-28-oic acid and tormentic acid showing particularly strong upregulation (**Figure 7A**). This pattern reinforces DY’s unique phytochemical profile centered on these compounds. The contrast between the cultivated GC and SG (both belong to *A. chinensis*) genotype showed that the SG variety consistently displayed higher levels of most ursane-type triterpenes, including ursolic acid and corosolic acid, whereas its cultivated counterpart GC showed a general suppression of this pathway (**Figure 7B**). A similar trend was observed when comparing the two *A. arguta* cultivars, LC and DY (**Figure 7C**), where DY again demonstrated a higher abundance of these triterpenoid acids. Moreover, the comparison between LC and SG highlighted SG’s unique ability to accumulate specific ursane-type triterpenes than LC (**Figure 7D**). Collectively, these targeted heatmaps crystallize the identification of ursane-type triterpenes as key metabolic differentiators, with the DY and SG varieties representing potent genetic resources for these compounds.

To evaluate the fold change differences of terpenoids between genotypes, a pairwise comparative analysis was conducted. The results revealed that DY variety exhibited a pronounced upregulation of triterpenoid acids compared to GC, WL, and SG (**Table 1**). Key markers such as 1-oxo-siaresinolic acid, camellisin B, and siegesbeckic acid were consistently elevated by over 400-fold (Log_2_FC > 8.7) in these comparisons. Conversely, DY showed an obvious downregulation of seco-iridoid glucosides like schizonepetoside A and geniposide when compared to SG and GC, with Log_2_FC -8.79 (**Table 1**). The LC variety also displayed a significant upregulation of diverse terpenoids such as dehydroisoactinidic acid (294-fold up) and dihydrocatalpol (171-fold up) compared to GC and SG. However, like DY, LC was characterized by very low levels of certain glucosylated terpenoids, including blumenol C glucoside (Log_2_FC -7.1) and vogeloside (Log_2_FC -4.84) as compared to SG. The SG was uniquely enriched in a range of compounds including geniposide (442-fold up) and several apocarotenoids (Log_2_FC > 7) (such as 3-Hydroxy-13-Apo-ϵ-Caroten-13-one, blumenol C glucoside; byzantionoside B) and phenolic-terpenoid conjugates like 3’-O-Caffeoyl sweroside Log_2_FC > 6.7, which were nearly absent or much lower in WL (**Table 1**). This suggests that the SG variety possesses a specialized metabolic profile distinct from the wild type.

**Table 1:** Significantly altered (VIP > 1.12 and Log_2_FC > 4.5 or > -3.1) terpenoids in pairwise comparisons between kiwifruit varieties.

Serial No.	Compounds	DY vs GC				
		VIP	P-value	Fold change	Log_2_FC	Type
1	1-Oxo-Siaresinolic acid*	1.16	0.01	680.90	9.41	up
2	Siegesbeckic acid	1.13	0.24	449.91	8.81	up
3	Camellisin B	1.15	0.07	427.01	8.74	up
4	2α,3α,23-Trihydroxyurs-12,20(30)-dien-28-oic acid*	1.16	0.02	366.36	8.52	up
5	7-Hydroxy-costol-malonyl glucoside	1.16	0.00	352.41	8.46	up
6	16,23:16:24-Diepoxycycloart-7-Ene-3,12,15,25-Tetrol	1.16	0.01	305.04	8.25	up
7	2,3,23-Trihydroxyolean-12,18(19)-dien-28-oic acid-glucoside	1.13	0.11	0.04	-4.58	down
8	Goshonoside F3	1.15	0.05	0.04	-4.83	down
9	2,3,19,23-Tetrahydroxyurs-12-en-28-oic acid-28-O-glucoside	1.14	0.07	0.02	-5.59	down
10	Schizonepetoside A	1.16	0.00	0.01	-6.89	down
11	Geniposide	1.15	0.04	0.00	-7.88	down
	Compounds	DY vs SG				
		VIP	P-value	Fold change	Log_2_FC	Type
12	Camellisin B	1.16	0.07	427.01	8.74	up
13	6’-O-β-D-glucosylbarlerin*	1.17	0.01	238.24	7.90	up
14	methyl (1S,4S,5R,6S,7S,8R,11S,12R,14S,15R)-12-acetyloxy-4,7-dihydroxy-6-[(1S,2S,6S,8S,9R,11S)-2-hydroxy-11-methyl-5,7,10-trioxatetracyclo[6.3.1.02,6.09,11]dodec-3-en-9-yl]-6-methyl-14-(3-methylbutanoyloxy)-3,9-dioxatetracyclo[6.6.1.01,5.011,15]pentadecane-11-carboxylate	1.17	0.04	178.47	7.48	up
15	6’-O-α-D-galactosylbarlerin*	1.17	0.00	140.17	7.13	up
16	Dehydroisoactinidic Acid	1.17	0.01	71.10	6.15	up
17	9-Hydroxy-8(14),15-Isopimaradien-1-one*	1.17	0.01	64.86	6.02	up
18	Geniposide	1.17	0.01	0.00	-8.79	down
19	Schizonepetoside A	1.17	0.00	0.01	-7.54	down
20	7-Deoxyloganate	1.17	0.01	0.01	-6.74	down
21	Elaeocarpucin F	1.17	0.00	0.03	-5.10	down
	Compounds	DY vs WL				
		VIP	P-value	Fold change	Log_2_FC	Type
22	1-Oxo-Siaresinolic acid*	1.17	0.01	680.90	9.41	up
23	Siegesbeckic acid	1.14	0.24	449.91	8.81	up
24	Camellisin B	1.16	0.07	427.01	8.74	up
25	2α,3α,23-Trihydroxyurs-12,20(30)-dien-28-oic acid*	1.16	0.02	366.36	8.52	up
26	2-Carboxy-3-hydroxy-A(1)-norlupan-20(29)-en-28-oic acid (Ceanothic acid)	1.17	0.01	249.30	7.96	up
27	Methyl 3,7-dihydroxy-15-cycloneranate	1.17	0.00	167.15	7.39	up
28	2,3,19-Trihydroxyolean-12-en-23,28-dioic acid	1.15	0.10	117.27	6.87	up
29	7-ethenyl-6-methoxy-4b-methyl-2,3,4,4a,5,6,7,8,8a,9-decahydro-1H-phenanthren-2-ol	1.17	0.00	103.39	6.69	up
30	Rhamnosylfucose	1.17	0.00	95.21	6.57	up
31	Gentiolactone	1.15	0.04	0.06	-4.01	down
32	Curcumenol	1.12	0.08	0.09	-3.40	down
33	Corymbolone	1.17	0.00	0.12	-3.10	down
	Compounds	LC vs GC				
		VIP	P-value	Fold change	Log_2_FC	Type
34	Dehydroisoactinidic Acid	1.16	0.04	294.10	8.20	up
35	1α,2α,3α,20β-tetrahydroxyurs -13(18)-en-28-oic acid*	1.16	0.01	175.28	7.45	up
36	1’S,4’S-4’-Dihydroabscisic acid-4’-O-β-glucopyranoside	1.17	0.01	174.35	7.45	up
37	Dihydrocatalpol	1.17	0.00	171.07	7.42	up
38	3β,6β,19α,24-Tetrahydroxyurs-12-en-28-oic acid*	1.16	0.01	156.49	7.29	up
39	2,3-Dihydroxyurs-12,18-dien-28-oic acid (Goreishic Acid I)	1.16	0.02	155.90	7.28	up
40	2,3,19-Trihydroxyolean-12-en-23,28-dioic acid	1.16	0.02	146.52	7.19	up
41	2,3,19-Trihydroxy-24-oxo-olean-12-en-28-oic acid	1.17	0.00	133.91	7.07	up
42	30-Hydroxyfraxinellone	1.16	0.01	133.74	7.06	up
43	16,23:16:24-Diepoxycycloart-7-Ene-3,12,15,25-Tetrol	1.17	0.00	106.94	6.74	up
44	2,3,19,23-Tetrahydroxyurs-12-en-28-oic acid*	1.17	0.00	100.98	6.66	up
45	2α,3α,20β,24-tetrahydroxyurs-13(18)-en-28-oic acid*	1.17	0.00	98.07	6.62	up
46	Blumenol C glucoside; Byzantionoside B	1.16	0.02	0.01	-7.41	down
47	2-[(1-hydroxy-2-methylpropan-2-yl)oxy]-6-(hydroxymethyl)oxane-3,4,5-triol	1.17	0.00	0.01	-7.18	down
48	2,3,19,23-Tetrahydroxyurs-12-en-28-oic acid-28-O-glucoside	1.15	0.07	0.02	-5.59	down
49	Goshonoside F3	1.16	0.05	0.04	-4.83	down
50	(6R,9R)-3-Oxo-α-ionol-β-D-glucoside	1.16	0.05	0.04	-4.64	down
51	2,3,23-Trihydroxyolean-12,18(19)-dien-28-oic acid-glucoside	1.14	0.11	0.04	-4.58	down
	Compounds	LC vs SG				
		VIP	P-value	Fold change	Log_2_FC	Type
52	Dehydroisoactinidic Acid	1.16	0.04	294.10	8.20	up
53	Dihydrocatalpol	1.17	0.00	171.07	7.42	up
54	2,3,19-Trihydroxy-24-oxo-olean-12-en-28-oic acid	1.17	0.00	133.91	7.07	up
55	6’-O-β-D-glucosylbarlerin*	1.17	0.00	54.76	5.77	up
56	methyl (1S,4S,5R,6S,7S,8R,11S,12R,14S,15R)-12-acetyloxy-4,7-dihydroxy-6-[(1S,2S,6S,8S,9R,11S)-2-hydroxy-11-methyl-5,7,10-trioxatetracyclo[6.3.1.02,6.09,11]dodec-3-en-9-yl]-6-methyl-14-(3-methylbutanoyloxy)-3,9-dioxatetracyclo[6.6.1.01,5.011,15]pentadecane-11-carboxylate	1.16	0.02	53.07	5.73	up
57	Paeoniflorin	1.16	0.01	51.72	5.69	up
58	6’-O-α-D-galactosylbarlerin*	1.17	0.00	37.48	5.23	up
59	Hastatoside*	1.17	0.00	35.76	5.16	up
60	Shizukafuranol	1.17	0.01	35.38	5.14	up
61	Blumenol C glucoside; Byzantionoside B	1.17	0.00	0.01	-7.10	down
62	Elaeocarpucin F	1.17	0.00	0.03	-5.10	down
63	8-(Beta-D-Glucopyranosyloxy)-2,7-Dimethyl-2,4-Decadienedioic Acid	1.17	0.00	0.03	-5.08	down
64	(6R,9R)-3-Oxo-α-ionol-β-D-glucoside	1.16	0.02	0.03	-5.03	down
65	Tanzawaic Acid B*	1.16	0.02	0.03	-4.96	down
66	Vogeloside	1.16	0.05	0.03	-4.84	down
	Compounds	SG vs WL				
		VIP	P-value	Fold change	Log_2_FC	Type
67	Geniposide	1.15	0.01	441.92	8.79	up
68	(5E,7E)-3,8,12-trimethyltrideca-5,7,11-triene-3,4-diol	1.15	0.00	165.27	7.37	up
69	3-Hydroxy-13-Apo-ϵ-Caroten-13-one	1.15	0.00	156.83	7.29	up
70	Blumenol C glucoside; Byzantionoside B	1.15	0.00	137.58	7.10	up
71	3’-O-Caffeoyl sweroside	1.15	0.00	107.73	6.75	up
72	7-ethenyl-6-methoxy-4b-methyl-2,3,4,4a,5,6,7,8,8a,9-decahydro-1H-phenanthren-2-ol	1.15	0.00	100.69	6.65	up
73	JiangxiBaiyingsu I	1.15	0.00	68.46	6.10	up
74	Rubochingoside G	1.15	0.00	56.05	5.81	up
75	17beta-Hydroxy-2-oxa-5alpha-androstan-3-one	1.15	0.00	55.34	5.79	up
76	3,13,15-Trihydroxyoleanane-12-one	1.15	0.01	50.77	5.67	up
77	Methyl 3,7-dihydroxy-15-cycloneranate	1.15	0.01	50.36	5.65	up
78	4,8a-dimethyl-1,2,3,4,4a,7,8,8a-octahydronaphthalen-4a-ol	1.15	0.00	46.68	5.54	up
79	3-Oxo-Alpha-Ionol 3’-(6’’-Malonyl)Glucoside	1.14	0.04	0.05	-4.45	down
80	ent-3α,16β,17,19-Tetrahydroxykauran-19-ylacetate-17-O-β-D-glucopyranoside	1.15	0.00	0.03	-5.27	down
81	(6R,9R)-3-Oxo-α-ionol-β-D-malonyl-glucoside	1.15	0.01	0.02	-5.53	down
82	methyl (1S,4S,5R,6S,7S,8R,11S,12R,14S,15R)-12-acetyloxy-4,7-dihydroxy-6-[(1S,2S,6S,8S,9R,11S)-2-hydroxy-11-methyl-5,7,10-trioxatetracyclo[6.3.1.02,6.09,11]dodec-3-en-9-yl]-6-methyl-14-(3-methylbutanoyloxy)-3,9-dioxatetracyclo[6.6.1.01,5.011,15]pentadecane-11-carboxylate	1.14	0.04	0.01	-6.12	down
83	Neohancoside B	1.15	0.02	0.01	-6.40	down

An asterisk (*) following a compound name denotes an isomer. LC (*Actinidia arguta* ‘Maolvfeng’, Dandong), DY (*Actinidia arguta*, Danyang flat kiwifruit, Dandong), GC (*Actinidia chinensis* ‘Guichang’, Zhaotong), SG (*Actinidia chinensis* ‘SunGold’, New Zealand), and WL (*Actinidia chinensis*, Wild type, Zhaotong).

For each compound, the VIP score, p-value, Fold Change, Log2-transformed Fold Change (Log_2_FC), and regulation type (up: higher in the first-named variety; down: lower in the first-named variety) are provided.

### 3.7 DPPH radical scavenging capacity

**Figure 8:**
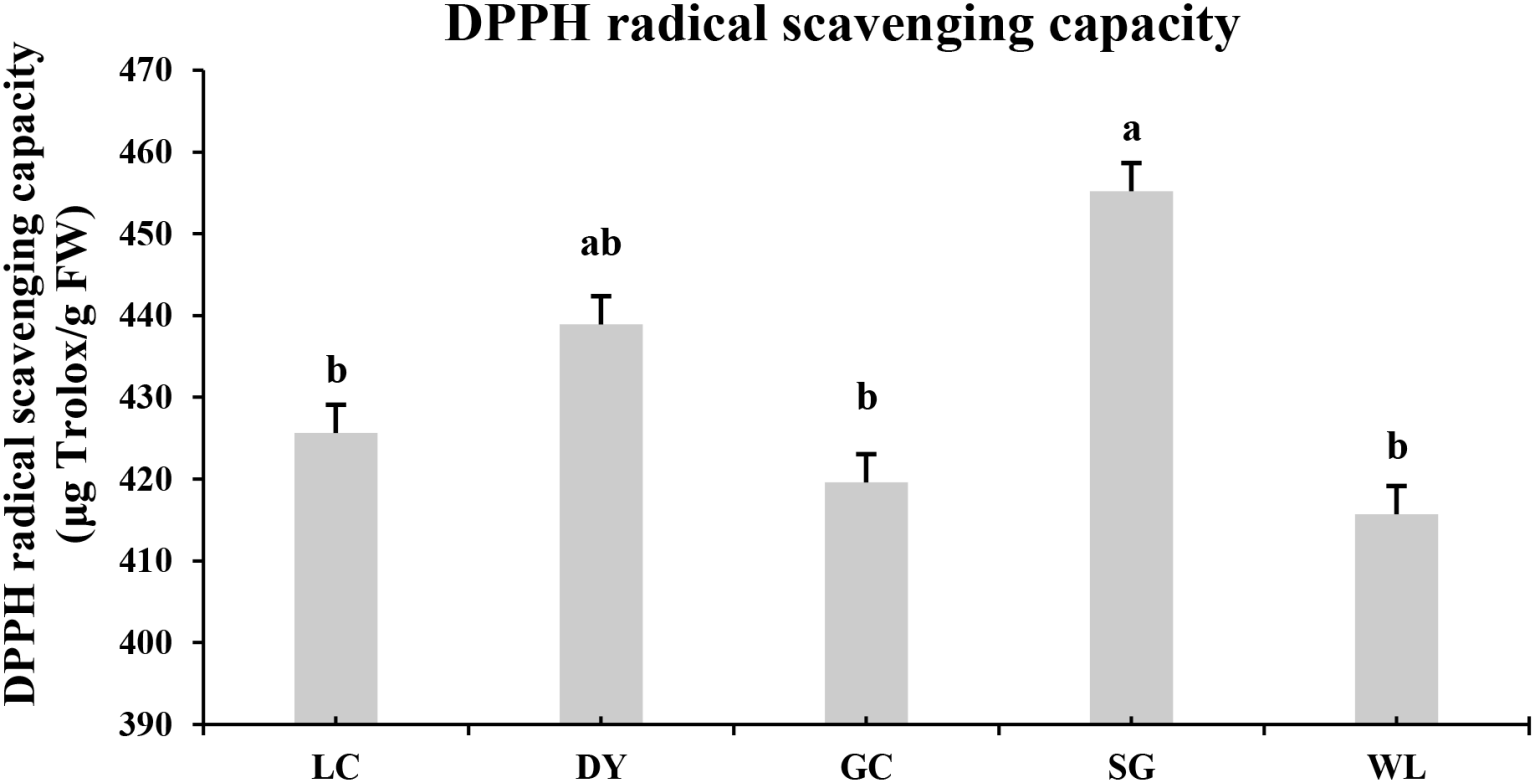
DPPH radical scavenging capacity in different kiwifruit varieties. Values are expressed as mean ± standard error (SE) (n=3). Different lowercase letters above the bars indicate significant differences according to Fisher’s Least Significant Difference (LSD) test (p< 0.05). LC (*Actinidia arguta* ‘Maolvfeng’, Dandong), DY (*Actinidia arguta*, Danyang flat kiwifruit, Dandong), GC (*Actinidia chinensis* ‘Guichang’, Zhaotong), SG (*Actinidia chinensis* ‘SunGold’, New Zealand), and WL (*Actinidia chinensis*, Wild type, Zhaotong).

The antioxidant capacity significantly varied among the five kiwifruit varieties (**Figure 8**). The SG exhibited the highest mean antioxidant capacity (455.20 ± 3.12 µg TE/g FW), followed by the Danyang (DY) variety (438.91 ± 3.43 µg TE/g FW) (**Figure 8**). The LC, GC, and WL fruits showed statistically similar, but lower, antioxidant capacity, with values of 425.63, 419.60, and 415.70 µg TE/g FW, respectively. These results indicate that the SG and DY kiwifruit varieties possess a superior *in vitro* antioxidant potential, which may contribute to their enhanced nutritional value and shelf-life.

## 4 Discussion

This study provides a comprehensive comparative analysis of terpenoid profiles, sugar content, and antioxidant capacity across five distinct kiwifruit varieties, representing both *Actinidia chinensis* and *A. arguta*. Our findings reveal profound chemical diversity influenced by genotype. They directly address how terpenoid profiles vary significantly among varieties and how genetic background dictates the accumulation of specific terpenoid classes. The identification of 309 distinct terpenoids significantly expands the known phytochemical landscape of kiwifruit (**Supplementary Table S1**). Previous research primarily focused on primary metabolites and the renowned ascorbic acid content in *Actinidia* species (Yu et al., 2020; Zhang et al., 2020; Li et al., 2024). The diversity and abundance of terpenoids uncovered here underscore their potential as major contributors to the fruit’s sensory and bioactive properties (**Figures 2**, **3**, **7**). The predominance of triterpenes and sesquiterpenoids aligns with findings in other fleshy fruits, where these non-volatile compounds often accumulate in the peel and flesh, contributing to defense mechanisms and potential health benefits (Acquavia et al., 2021; Rao et al., 2021b, a; Saini et al., 2022; Duan et al., 2025b, a). The clear separation of varieties through PCA, with high intra-variety correlation, robustly confirms that the terpenoid metabolome serves as a potent chemotaxonomic marker. This capability to distinguish kiwifruit genotypes is similar to how volatile terpenes are used as markers for citrus and grape varieties (Acquavia et al., 2021; Saini et al., 2022).). Overall, these results provide a valuable foundation for germplasm characterization in breeding programs.

The observed varietal differences in terpenoid accumulation likely stem from genetic variations in the terpenoid biosynthetic pathway. Terpenoids are synthesized via the methylerythritol phosphate (MEP) and mevalonate (MVA) pathways, leading to the production of universal precursors that are subsequently modified by terpene synthases (TPS) and cytochrome P450s (CYP450s). In kiwifruit, the TPS gene family has been partially characterized, and certain members, such as AcTPS1b, are known to produce specific terpenes like 1,8-cineole (Zeng et al., 2020; Wang et al., 2023). The distinct terpenoid fingerprints of DY and SG may be attributed to differential expression or activity of key TPS and CYP450 genes, which could be regulated by transcription factors and phytohormones. Future studies focusing on the transcriptomic and enzymatic levels of these pathways in the studied varieties would elucidate the genetic and biochemical mechanisms underlying the observed metabolic diversity.

The hierarchical clustering and pairwise comparisons revealed distinct, variety-specific terpenoid fingerprints with significant implications for sensory and nutritional quality (**Figures 2**, **4**; **Table 1**). The DY (*A. arguta*) pronounced enrichment in ursane-type triterpenes, such as pomolic acid and its derivatives, is particularly noteworthy (**Figure 5**; **Table 1**). Ursane-type triterpenes are synthesized via the cyclization of 2,3-oxidosqualene by oxidosqualene cyclases (OSCs), followed by oxidation by CYP450s and decoration by glycosyltransferases (Tholl, 2015; Lu et al., 2016; Holopainen et al., 2025). The high abundance of these compounds in DY suggests an upregulation of these enzymatic steps in its triterpenoid biosynthesis. These compounds are well-documented in other plant sources like apples and *Ilex chinesis* for their anti-inflammatory and hepatoprotective activities (Cargnin and Gnoatto, 2017; Chan et al., 2023; Shao et al., 2023; Khwaza and Aderibigbe, 2024). The high accumulation of these bioactive triterpenes in DY marks it as a prime candidate for breeding programs aimed at enhancing the nutraceutical value of kiwifruit, particularly within the *A. arguta* gene pool. Similarly, the co-clustering of the WL and LC varieties, characterized by their high accumulation of oleanane-type triterpenes such as alphitolic acid, demonstrates that certain metabolic patterns can transcend species boundaries, potentially representing ancestral traits. These compounds often retain in wild genotypes, can contribute to defensive or astringent properties (Pan et al., 2023; Sakna et al., 2023). The significant metabolic shift observed between the wild (WL) and cultivated GC (both *A. chinensis*), marked by a general suppression of complex triterpenoid acids in GC (**Figure 3**), suggests that possible breeding for enhanced palatability has indirectly selected against this metabolic pathway. This reflects a broader trend of attenuating potentially undesirable, bitter-tasting compounds to improve fruit quality (Ghosh, 2018; Sakna et al., 2023), a key trade-off in the selection process observed in other fruit crops such as in apples and tomatoes (Whitehead and Poveda, 2019; Salazar-Mendoza et al., 2023).

A key finding with direct application for breeding is the superior antioxidant capacity of the SG (*A. chinensis*) and DY (*A. arguta*) genotypes, as measured by the DPPH assay (**Figure 8**). While this enhanced activity cannot be attributed to a single compound class, it coincides with their high overall terpenoid abundance (Rao et al., 2021b; El-Saadony et al., 2024; Li et al., 2024). As DPPH radical scavenging is influenced by multiple phytochemical classes (e.g., phenolics, or other bioactive compounds), the antioxidant capacity measured here represents a composite effect. Triterpenic acids like ursolic acid and oleanolic acid, which were significantly upregulated in SG and DY (**Figure 7**; **Table 1**), have established potent antioxidant properties *in vitro* and *in vivo* (JC Furtado et al., 2017; Castellano et al., 2022; Günther and Bednarczyk-Cwynar, 2025). The antioxidant activity of ursane-type triterpenes is structurally attributed to the presence of hydroxyl groups, especially at the C-3 position, and the carboxylic acid moiety, which can donate hydrogen atoms and stabilize free radicals. For instance, ursolic acid and pomolic acid, both abundant in DY, possess multiple hydroxyl groups that enhance their radical scavenging capacity. Similarly, the seco-iridoid geniposide in SG contains a glycosylated moiety that may influence its antioxidant potential. Apocarotenoids, such as those found in SG, are known for their antioxidant properties due to the conjugated double bond system that can delocalize unpaired electrons. This suggests that terpenoids are significant contributors to the overall antioxidant network in kiwifruit, working synergistically with the well-known vitamin C and phenolic compounds (Zhang et al., 2020; Li et al., 2024; Moysidou et al., 2024). Therefore, selecting high-terpenoid varieties like SG and DY presents a viable breeding strategy for enhancing the nutraceutical value of kiwifruit, offering consumers a product with superior free-radical scavenging potential (Moysidou et al., 2024).

The commercial SG (*A. chinensis*) showed unique enrichment in seco-iridoid glucosides like geniposide and specific apocarotenoids, compounds nearly absent in the wild type WL, pointing to a highly specialized biosynthetic pathway established through selective breeding. Seco-iridoids are derived from the terpenoid pathway via iridoid synthase, while apocarotenoids are produced through oxidative cleavage of carotenoids by carotenoid cleavage dioxygenases (CCDs). The unique accumulation of these compounds in SG suggests a rewiring of these specific branches of terpenoid metabolism during domestication. Geniposide, known from *Gardenia jasminoides*, exhibits various bioactivities, including neuroprotective effects (Birekdar, 2025). Apocarotenoids, derived from carotenoid degradation, are crucial aroma volatiles in fruits like saffron and passion fruit (Siracusa et al., 2011; Simkin, 2021; Ahrazem et al., 2022). Their abundance in SG may underpin unique aromatic notes that contribute to its commercial success. Furthermore, the very high soluble sugar content of SG highlights a successful breeding focus on palatability. Critically, our results demonstrate that this “sweetness” is not mutually exclusive with a rich and unique terpenoid profile. Instead, it can redirect metabolic flux towards different, valuable specialized compounds (Litvinov et al., 2021), as evidenced by SG’s distinct phytochemical signature. The combination of exceptionally high sugar and high terpenoid content in ‘SunGold’ presents a valuable model for breeding, demonstrating that intense selection for sweetness does not necessarily compromise the production of diverse specialized metabolites.

Our findings showed that terpenoid profiles are strongly associated with genetic background, answering a central question of this study. The resulting varietal signatures have profound implications for flavor, nutrition, and bioactivity. The clear contrast between wild and cultivated varieties from the same region underscores the impact of human selection on the fruit’s phytochemical composition and highlights the potential metabolic cost of breeding for consumer-preferred traits. Conversely, the comparison between the high-performing SG (*A. chinensis*) and DY (*A. arguta*) shows that superior terpenoid content and antioxidant capacity are achievable breeding goals across different species. Moving forward, this detailed metabolic map provides a foundational resource for targeted breeding programs. The identification of key marker terpenoids associated with high antioxidant capacity (e.g., ursane-type triterpenes in DY) or unique flavors (e.g., seco-iridoids in SG) offers tangible biochemical targets for marker-assisted selection. Future work should focus on correlating these specific terpenoid profiles with sensory analysis and investigating their bioavailability. By integrating the metabolomic data presented here with genomic tools, we can accelerate the development of next-generation kiwifruit cultivars that optimally balance superior taste with enhanced health-promoting properties, directly addressing the goals of modern kiwifruit breeding.

This study has inherent limitations, as genetic background is confounded with geographical and agronomic factors. Although strong genotype-specific clustering supports a primary genetic driver for terpenoid profiles, environmental influences cannot be ruled out. Our work thus provides a detailed phenotypic map and establishes a robust variety-terpenoid association, forming an essential foundation for causal investigation. Being descriptive, it profiles the metabolic “what” rather than the genetic “why.” To move from correlation to mechanism, future research must integrate genomic, transcriptomic, and enzymological data with these metabolomic findings to pinpoint the regulators of key pathways, such as ursane-type triterpene synthesis in ‘Danyang’ or seco-iridoid production in ‘SunGold.’ Targeted validation through multi-location trials and specific metabolite-function correlation analyses is also needed. These strategies will convert the identified metabolic markers into precise tools for breeding kiwifruit cultivars with optimized flavor, nutrition, and resilience.

## 5 Conclusions

This study reveals distinct, genotype-associated terpenoid profiles in kiwifruit, with significant variation occurring both between major species (*Actinidia chinensis* vs. *A. arguta*) and among cultivated varieties within them. The identification of 309 terpenoid compounds provides a broad view of the phytochemical potential within the kiwifruit germplasm. Crucially for breeders, we found that desirable traits like high terpenoid content and superior antioxidant capacity are not confined to a single species, being prominently exhibited by the *A. arguta* cultivar ‘Danyang’ (DY) and the *A. chinensis* cultivar ‘SunGold’ (SG). The stark metabolic contrast between the wild *A. chinensis* (WL) and its cultivated counterpart GC illustrates a common trade-off in modern selection, where breeding for palatability can lead to the reduction of certain bioactive triterpenoids. Conversely, the profile of ‘SunGold’ (SG) serves as a benchmark for successful cultivar development, proving that intense selection for sweetness and aesthetic qualities can be synergistically combined with a unique and abundant terpenoid profile, including valuable seco-iridoids and apocarotenoids. Collectively, our findings deliver a terpenoid roadmap and a set of metabolic markers for kiwifruit breeding. This resource empowers breeders to make informed choices by selecting parental lines from across the genetic spectrum to develop the next generation of cultivars that strategically balance superior sensory appeal with enhanced, targeted health-promoting properties.

## Data Availability

The original contributions presented in the study are included in the article/**Supplementary Material**. Further inquiries can be directed to the corresponding authors.
