# Supplementary material for: Comparative metabolomics identifies enhanced ursane-type triterpenoids and antioxidant capacity in Actinidia arguta ‘Danyang’ kiwifruit
Source: Front Plant Sci. 2026 Feb 16;17:1771986. doi: 10.3389/fpls.2026.1771986 (PMC12950792; doi:10.3389/fpls.2026.1771986)
Supplement: Supplementary file 1 [file Table1.docx]

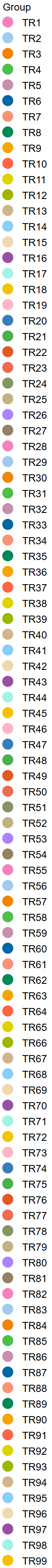

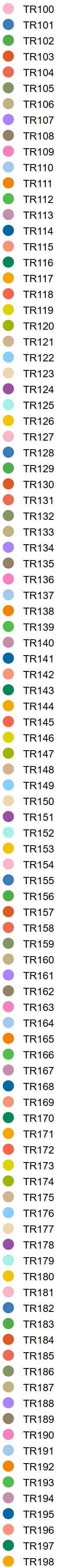


**Figure S1.** Annotated color key for terpenoid compounds in Figure 2B. Visual representation of the color-coded terpenoid compounds (TR1-TR199) analyzed in the compound-wise PCA.
